# Isocaloric High-Intensity Interval and Circuit Training Increases Excess Post-Exercise Oxygen Consumption and Lipid Oxidation Compared to Moderate-Intensity Continuous Training

**DOI:** 10.3390/sports13100355

**Published:** 2025-10-06

**Authors:** Viviane Faleiro, Alexandre V. Gurgel, Thiago T. Guimarães, Tiago C. Figueiredo, Felipe G. Teixeira, Bruno Jotta, Estêvão R. Monteiro, Alexandre G. Meirelles, Carla C. A. Caldas, Maicon T. de Almeida, Raquel C. Castiglione, Silvio R. Marques-Neto

**Affiliations:** 1 Programa de Pós-graduação em Ciências da Atividade Física, Universidade Salgado de Oliveira (UNIVERSO), Niterói 24030-060, Brazil; vivivolei87@hotmail.com (V.F.); coachgurgel@gmail.com (A.V.G.); thiago.guimaraes@nt.universo.edu.br (T.T.G.); carlaade@hotmail.com (C.C.A.C.); maicon_teixeira_almeida@hotmail.com (M.T.d.A.); 2 Laboratório de Fisiologia do Exercício (LAFIEX), Universidade Estácio de Sá, Rio de Janeiro 20771-004, Brazil; tc-figueiredo@uol.com.br (T.C.F.); fegute1@hotmail.com (F.G.T.); brunojottac@mail.com (B.J.); 3 Graduate Program in Rehabilitation Science (PPGCR/UNISUAM), Centro Universitário Augusto Motta, Rio de Janeiro 21032-060, Brazilalexandremeirelles@souunisuam.com.br (A.G.M.); 4 Graduate Program in Biopsycosocial Health, Centro Universitário Augusto Motta (PPGBS/UNISUAM), Rio de Janeiro 21032-060, Brazil; 5 Laboratório de Pesquisas Clínicas e Experimentais em Biologia Vascular (BioVasc), Universidade do Estado do Rio de Janeiro (UERJ), Rio de Janeiro 20550-013, Brazil; rccastiglione@gmail.com

**Keywords:** moderate-intensity continuous training, high-intensity interval training, high-intensity circuit training, excess post-exercise oxygen consumption, energy expenditure, substrate oxidation

## Abstract

Background: This study compared energy expenditure (EE), substrate metabolism, and excess post-exercise oxygen consumption (EPOC) during moderate-intensity continuous training (MICT), high-intensity interval training (HIIT), and high-intensity circuit training (HICT) isocaloric sessions. Methods: Twelve trained male participants completed isocaloric exercise sessions equalized for EE and average power (AP) across the three modalities. Postexercise EE, carbohydrate and lipid oxidation rates, and EPOC were measured 30 and 60 min after training. Results: Total EE and AP during exercise were similar between the protocols. However, EPOC was significantly higher for HIIT (319.0 ± 88.03 mL) and HICT (329.1 ± 27.79 mL) than for MICT (168.5 ± 21.84 mL), demonstrating greater post-exercise metabolic demand in high-intensity protocols. At 30 min post-exercise, carbohydrate oxidation remained elevated in the HIIT (3.70 ± 1.04 mg/kg/min) and HICT (4.06 ± 1.03 mg/kg/min) groups compared to that in the MICT group (1.42 ± 0.58 mg/kg/min), while lipid oxidation rates were also higher (HIIT: 1.08 ± 0.41; HICT: 1.20 ± 0.24 mg/kg/min; MICT: 0.61 ± 0.20 mg/kg/min). These effects persisted for 60 min, with HIIT and HICT maintaining significantly greater carbohydrate and lipid oxidation than MICT. Correlation analysis indicated a strong relationship between carbohydrate oxidation during exercise and lipid oxidation after 60 min of exercise. Conclusions: High-intensity protocols (HIIT and HICT) promote prolonged postexercise EE, enhance carbohydrate and lipid oxidation, and optimize metabolic recovery, making them effective strategies for maximizing energy utilization beyond the training session.

## 1. Introduction

Since 1980, there has been an increased global focus on strategies for effective weight loss and long-term weight maintenance, given the challenges faced by approximately one-third of the global population in achieving sustainable body weight management [1]. Successful weight loss and weight maintenance significantly contribute to improved health outcomes by reducing the risk of chronic conditions, including type 2 diabetes mellitus, cardiovascular diseases, and several cancers, thereby enhancing both life expectancy and overall quality of life [2]. Regular physical exercise is widely recognized as one of the most effective nonpharmacological approaches for achieving sustainable weight loss and maintaining optimal body composition. Exercise primarily contributes to these outcomes by increasing lean body mass (LBM) and total energy expenditure (EE), leading to sustained reductions in fat mass (FM) over time [3]. EE, which includes basal metabolic rate (BMR) and energy expenditure during and after physical activity, is fundamental for successful and lasting weight control and metabolic health [4].

Current physical activity guidelines advocate 150–300 min of moderate-intensity or 75–150 min of vigorous-intensity aerobic exercise weekly [5]. However, total EE and substrate utilization during and after exercise are significantly influenced by exercise intensity, volume, and mode [6]. Typically, high-intensity exercise promotes greater EE and increases carbohydrate mobilization during exercise, whereas low- to moderate-intensity exercise primarily mobilizes lipids [7].

Post-exercise energy metabolism is closely tied to substrate mobilization during exercise, and evidence indicates that high-intensity exercise significantly depletes glycogen reserves, thereby enhancing lipid oxidation during recovery [8]. Nonetheless, the precise impact of substrate utilization during different exercise modalities on post-exercise EE and substrate oxidation remains underexplored, especially in isocaloric training sessions. It is well established that substrate utilization during exercise depends on exercise intensity. At low intensities (<40% VO_2_max), fat is the predominant substrate, particularly during prolonged exercise (>90 min) when glycogen stores are depleted. At moderate intensities (40–65% VO_2_max), carbohydrate and fat contributions are more balanced, with fat oxidation peaking near the upper limit of this range. At higher intensities (≥70% VO_2_max), carbohydrate becomes the dominant energy source due to the rapid ATP demand. Importantly, individuals with greater aerobic capacity tend to shift the point of maximal fat oxidation to higher intensities. These concepts are central to interpreting the metabolic responses observed in the present study [9].

Research findings have shown inconsistencies in the influence of exercise modality on excess post-exercise oxygen consumption (EPOC). Intermittent exercises, such as high-intensity interval training (HIIT), typically produce a higher EPOC than moderate-intensity continuous training (MICT), even when both are normalized by total EE. This increased EPOC observed in HIIT is primarily attributed to its substantially greater relative intensity, despite isocaloric normalization by EE, highlighting the significant differences in metabolic demands and adaptations elicited by varying intensities within isocaloric comparisons [10]. For instance, treadmill running demonstrated a higher EPOC than cycling at moderate intensities under isocaloric conditions, whereas resistance training and HIIT showed comparable EPOC values [10].

Recently, high-intensity circuit training (HICT), which involves multi-joint functional movements and variable rest intervals, has garnered interest because of its potential to significantly enhance EE during and after exercise [11]. Although HICT and HIIT demonstrate similar EE profiles during and after exercise [12], potential differences in training load, relative intensity, and muscle involvement may distinctly affect postexercise metabolic responses. Therefore, the present study aimed to investigate and compare post-exercise EE and substrate utilization (specifically carbohydrate and lipid oxidation) after isocaloric sessions of HICT, HIIT, and MICT in healthy men. The major difference in this study lies in the fact that the isocaloric sessions were equalized by both EE and relative intensity values.

Although fat oxidation is suppressed during very intense exercise, post-exercise recovery is characterized by a compensatory increase in fat utilization. This shift reflects key recovery processes, such as phosphocreatine resynthesis, lactate clearance, oxygen store replenishment, thermogenesis, and hormonal activity, all of which contribute to elevated post-exercise oxygen consumption (EPOC). Stressing this metabolic shift is essential to understand the relevance of high-intensity protocols for recovery metabolism [13]. We hypothesized that substrate utilization patterns during different exercise modalities would significantly affect substrate mobilization, consequently influencing the EPOC and post-exercise EE. Furthermore, we anticipated that HICT and HIIT would lead to greater post-exercise metabolic activity and lipid oxidation than MICT, primarily because of their significantly higher relative intensities and greater engagement of muscle mass during the isocaloric exercise sessions.

## 2. Materials and Methods

### 2.1. Ethics and Experimental Design

The study was approved by the Salgado de Oliveira University (UNIVERSO) Ethics Committee (CAEE: 02469418.2.0000.5289) and conducted in accordance with RES 466/12 and 510/2016 of the Brazilian National Health Council and the 1964 Declaration of Helsinki. A counterbalanced, randomized, crossover, within-participant experimental design was used to compare postexercise metabolic profiles induced by HICT, HIIT, and MICT isocaloric exercise bouts in 12 healthy male participants. They visited the laboratory for four sessions over a seven-day period, with at least forty-eight hours between visits. During the first visit, anthropometric assessments and maximum cardiopulmonary exercise testing (CPET) were performed using a rowing ergometer [14].

During the second, third, and fourth visits, the participants completed three isocaloric exercises (HICT, HIIT, and MICT) in a randomized order (https://www.studyrandomizer.com/, accessed on 5 May 2023). Heart rate (HR) and gas exchange were continuously assessed before (10 min) and every 5 min after each training session for 60 min. In addition, the rating of perceived exertion (RPE) was applied and evaluated 10 min after the training sessions to monitor the internal training load. All sessions were held in the morning (07:00 and 11:00 a.m.) for each participant, under similar environmental conditions (~ 22 °C and ~ 60% humidity).

### 2.2. Participants

Twelve healthy male participants who were recreationally HICT-trained were recruited by convenience from professional teams to participate in this study, which was conducted at Salgado de Oliveira University. An a priori sample size calculation (effect size = 0.92; 1−β = 0.80; α = 0.05; nonsphericity correction = 1.0) using G*Power indicated that 12 participants would be adequate to achieve statistical power [15].

The inclusion criteria were as follows: (1) a minimum of three years of previous experience with HICT; (2) no neuropsychiatric, cardiovascular, or osteoarticular diseases; and (3) no use of any medication or ergogenic aids. Participants were excluded if they used any caffeinated or alcoholic beverages two days before each experimental session or performed any strenuous exercise two days before each experimental session.

During the data collection period (October to December 2024), the participants were instructed not to engage in any non-study HICT or HIIT programs. All participants were instructed to arrive after a standardized overnight fast (≥8 h), and the sessions were scheduled in the morning (07:00–11:00 a.m.). Participants were asked to replicate their last meal before each session and maintain a consistent dietary intake during the study period. Participants were advised to maintain their sleeping habits and avoid caffeine, ergogenic, and soothing consumption during data collection. Prior to the study, all participants were provided with verbal and written explanations of the study procedures, provided written informed consent, and completed the Physical Activity Readiness Questionnaire (PAR-Q).

### 2.3. Anthropometric Assessments

According to the available recommendations [16], the participants’ body mass and height were measured using a weighing scale and stadiometer (Filizola model 31, São Paulo, Brazil). Body composition was estimated based on body density and fat percentage. Skinfold thicknesses (chest, thigh, and abdomen) were assessed using BodyMetrix^®^ (IntelaMetrix, BodyMetrix™ BX-2000, Livemore, CA, EUA), an A-mode 2.5 MHz portable ultrasound device that emits high-frequency sound waves to penetrate body tissues. Ultrasonography was performed according to the manufacturer’s recommendations. The gel was applied to the wand head and placed perpendicular to the skin contact point at three observation sites. Local averaging of the measured signal was performed in small circles approximately 5 mm from the observation site for roughly 5 s.

### 2.4. Cardiopulmonary Exercise Test (CPET)

Maximal CPETs was performed on a Model-C rowing ergometer (Concept II, Inc., Morrisville) using the modified Conconi test [14]. Before starting the test, the participants were allowed to acclimatize for 10 min (~22 °C). Subsequently, they were allowed to warm up for 5 min with a 75 W power output (PO) intensity. The participants then began rowing at a self-selected cadence with a steady PO of 75 W for the first minute. The PO was increased by 25 W every 60 s until the participant was voluntarily exhausted. This load increment protocol gradually increased the corresponding HR by less than 8 b.min^−1^ each minute.

Gas exchange data were acquired breath-by-breath during rest and maximal CPETs via a PNOE system (ENDO Medical, Palo Alto, CA, USA). A face mask (Hans Rudolph^TM^, Kansas City, MO, USA) that covered the mouth and nose of the participants was attached to a bidirectional digital high-flow valve and fastened using mesh hairnet and Velcro straps. The gas analyzer and pneumotachograph were calibrated according to the manufacturer’s instructions.

Finally, the VO_2peak_ was computed as the highest VO_2_ value reached at the end of the test. In addition, heart rate was continuously monitored (Polar^®^ V800, Polar Electro, Oy, Kempele, Finland). The Concept II ergometer measured exercise intensity as PO (in watts). During all tests, the ergometers were calibrated according to the manufacturer’s instructions.

### 2.5. Determination of Ventilatory Threshold (VT) and Respiratory Compensation Point (RCP)

VT and RCP were determined during maximal CPETs using the criteria proposed by Marques-Neto et al. [17]. In this method, VT and RCP were detected automatically by searching for VE/VO_2_ and VE/VCO_2_ breakpoints, respectively. Briefly, breath-by-breath values for VE/VO_2_ and VE/VCO_2_ were fitted to a fifth-degree polynomial using a smooth spline curve using the least-squares method. The minima obtained from the first-order derivatives of the fitted polynomials for VE/VO_2_ and VE/VCO_2_ were used to calculate VT and RCP values, respectively. The same expert evaluator examined VT and RCP for each participant.

### 2.6. Calculation of Substrate Metabolism

EE, which is the EE induced by an exercise bout (e.g., exercise EE–resting EE), was calculated individually from VO_2_ and VCO_2_ in L·min^−1^ using the Weir Equation [18]:

EE (kcal) = [(3.941 × [average VO_2_]) + (1.106 × [average VCO_2_]) × time] (1)

In addition, EE was calculated individually for the 60 min recovery period. EE values were calculated as the total kcal and converted to kJ (1 kcal = 4.18 kJ).

Carbohydrate and lipid metabolism oxidation (g·min^−1^) were computed from VO_2_ (ml/kg/min^−1^) and VCO_2_ (ml/kg/min^−1^), respectively, using stoichiometric equations, assuming that protein oxidation during the training sessions was negligible [19].

Carbohydrate oxidation rate (mg/kg/min^−1^) = 4.585 × VCO_2_ − 3.226 × VO_2_ (2)

Lipid oxidation rate (mg/kg/min^−1^) = 1.695 × VO_2_ − 1.701 × VCO_2_ (3)

Carbohydrate energy output (cal/kg/min^−1^) = Carbohydrate oxidation rate × 4 (4)

Lipid energy output (cal/kg/min^−1^) = Lipid oxidation rate × 9 (5)

Total energy output (cal/kg/min^−1^) = Carbohydrate energy output (cal/kg/min^−1^) + Lipid energy output (cal/kg/min^−1^) (6)

Percentage of energy from lipid = Lipid energy output ÷ Total energy output (7)

Percentage of energy from lipid = Lipid energy output ÷ Total energy output (8)

EPOC was calculated by subtracting the area under the VO_2_ curve from the basal value in the post-exercise phase. For this purpose, a monoexponential model was fitted to characterize the VO_2_ decay kinetics, and the coefficient of determination (R^2^) was calculated to assess the goodness-of-fit of the model (Equation (7)).

VO_2_ (t) = EEVO_2_ − A1 (1 − e^−(t − TD)/t1^) (9)

where VO_2_ (t) is the whole-body VO_2_ at time t, A1 is the amplitude of the fast component, TD is the time delay, and t1 is the time constant.

### 2.7. Monitoring of Internal Training Load

RPE was determined using an adapted Borg scale, ranging from 0 to 10. The scale has mode-specific verbal and pictorial descriptors in numerical responses and a narrow range from 0 (extremely easy) to 10 (extremely difficult), as described by Foster [20]. The RPE method uses the simple question: “How was your workout today?” The use of this tool only confirmed that there was no difference in the perception of applied loads. The internal perceptual training load was calculated from RPE by multiplying RPE by the duration of the training session (LOAD = RPE × time).

### 2.8. Isocaloric Exercise Protocols

The isocaloric training sessions were equalized with the total EE during exercise. MICT sessions were performed using a rowing ergometer at 90% VT for 17 min. HIIT was also performed on a rowing ergometer, with six stimuli of 20 s at 100% RCP interspersed by six active recovery periods of 10 s at 80%VT (ratio 2:1) for a total duration of 12 min.

As previously recommended, HIIT prescriptions were established based on cardiopulmonary thresholds [21]. In this sense, the requirements of HIIT concerning work interval intensity and time as well as relief interval intensity and duration were met. The choice of ergometer rowing was based on its everyday use in HICT centers [22].

HICT was performed in the following order: deadlift, clean, snatch, thruster, front squat, and kettlebell swing. All exercises were performed for 20 s, with 25% body mass load, and interspersed with 10 s intervals between them. Two sets were performed for each exercise, with a total duration of 12 min. The participants were verbally encouraged, and the quality of the movement techniques was controlled based on the criteria published elsewhere.

The choice of the HICT protocol followed the following criteria: (a) similarity to the Tabata protocol concerning stimulus (20 s) and recovery (10) time, owing to the great popularity of the protocol [23], and (b) the load used was equalized to the participants’ body weight. The choice of load was to increase the external validity of our study because the equipment commonly found in HICT centers consists of Olympic lifting bars, kettlebells, medicine balls, and plyometric boxes [24].

### 2.9. Equalization of Training Overload

The average power (AP) generated during both the MICT and HIIT sessions on the Concept 2 ergometer was standardized in watts. In the case of HICT, the participants adhered to a prescribed pace to execute repetitions with a load equivalent to 25% of their body weight, allowing determination of the total training volume (kg × reps × series).

**Figure 1:**
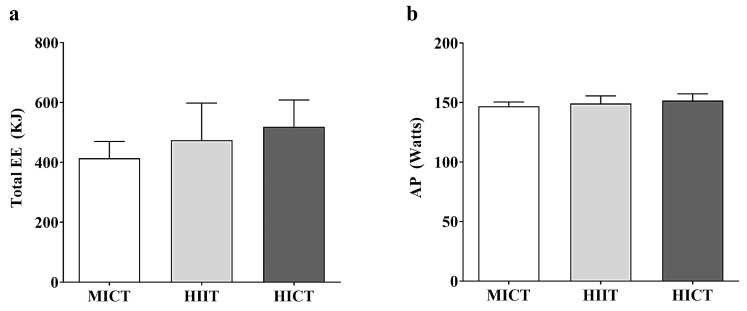
Comparison between (**a**) total training energy expenditure (EE) and (**b**) average power (AP) between MICT, HIIT, and HICT training sessions, where EE is expressed in kJ, and AP is expressed in watts.

The total training volume was initially established in kgm and subsequently converted to kgm.min^−1^ (kgm divided by time) and then to watts (kgm.min^−1^ divided by 6.12). This comprehensive process ensured that the total work for MICT, HIIT, and HICT was presented in watts (Figure 1b), which is consistent with the established methodology [24].

### 2.10. Statistical Analysis

Descriptive statistics are presented as mean ± standard deviation (SD) together with test statistics (F or t values), degrees of freedom (df), exact *p*-values, effect sizes (Cohen’s d for pairwise comparisons and partial η^2^ for ANOVA), and 95% confidence intervals (CI). Data normality was determined using the Shapiro–Wilk test, and all outcomes were normally distributed. The sphericity of the data was assessed using Mauchly’s test, and whenever sphericity was violated, the Greenhouse–Geisser correction factor was applied. G*Power software was used to calculate the appropriate sample size to detect differences in EPOC between protocols, based on a one-tailed t-test with the following parameters: *p*-value = 0.05, power = 0.80, and effect size = 0.92. Comparisons between means at an isolated time were performed using one-way analysis of variance (One-Way ANOVA) for dependent samples, and differences between groups were established using Tukey’s post hoc test.

Comparisons between means as a function of time (EPOC curve) were performed using two-way analysis of variance (Two-Way ANOVA), and differences between groups were established using Tukey’s post hoc test. The association between variables was quantified by calculating the Pearson’s correlation coefficient (r). The significance level was set at *p* < 0.05. Statistical analysis was performed using the Statistical Package for the Social Sciences (IBM SPSS Statistics 23, Corp., Armonk, NY, USA).

## 3. Results

The participants’ characteristics are presented in Table 1. These values reflect resistance-trained athletes with elevated lean body mass, which explains the coexistence of BMI in the overweight range with a low-fat percentage.

Data are presented as mean ± standard deviation. BMI, body mass index; VT, ventilatory threshold; RCP = Respiratory compensation point; VO_2_max, maximal oxygen uptake; HR, maximum heart rate.

### 3.1. Comparison Between Training Overload and Isocaloric Training Session

Figure 1 shows a comparison of EE (a) and AP (b) during the three isocaloric training sessions (MICT, HIIT, and HICT). No significant differences were observed in total EE and AP between the different training sessions.

### 3.2. EPOC During MICT, HIIT, and HICT

**Figure 2:**
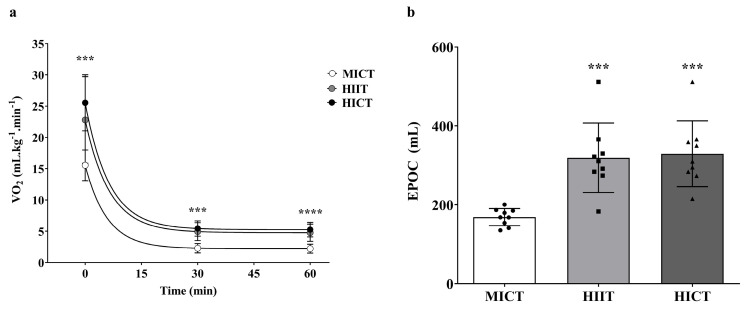
Parameters related to the quantification of excess oxygen consumption in the post-exercise phase (EPOC) during MICT, HIIT, and HICT training sessions. (**a**): Analysis of VO_2_ time course. (**b**) Analysis of the total amount of O_2_ mobilized during the 60 min period (AUC). *** *p* = 0.0002 vs. MICT. **** *p* = 0.0001 vs. MICT.

Figure 2a illustrates the temporal time course of VO_2_ following MICT, HIIT, and HICT sessions over 60 min post-training; the monoexponential fitting showed consistently high goodness-of-fit values, confirming the adequacy of the model in representing VO_2_ decay across all conditions (R^2^ = 0.82 for HIIT, R^2^ = 0.93 for HICT, and R^2^ = 0.95 for MICT). Notably, we observed that the VO_2_ values immediately after exercise (0 min) in the HIIT (22.78 ± 9.93 mL/kg/min^−1^) and HICT (25.52 ± 4.48 mL/kg/min^−1^) groups were significantly higher than those in the MICT group (15.53 ± 2.46 mL/kg/min^−1^; *p* < 0.001). At 30 min, HIIT (4.94 ± 1.43 mL/kg/min^−1^) and HICT (5.43 ± 1.22 mL/kg/min^−1^) showed significantly higher VO_2_ values than MICT (2.29 ± 0.75 mL/kg/min^−1^; *p* < 0.001), and they remained significantly elevated until 60 min (*p* < 0.001).

To quantify the total amount of oxygen mobilized during the post-exercise period (EPOC), we applied the area under the curve (AUC) method (Figure 2b). EPOC differed significantly across conditions (ANOVA: F(2,22) = 14.39, *p* < 0.0001, partial η^2^ = 0.53). Post hoc analyses revealed greater EPOC in HIIT (319.0 ± 88.0 mL O_2_) compared to MICT (168.5 ± 21.84 mL of O_2_) (mean difference = 150.5 mL O_2_, 95% CI of difference [66.7–234.2]; t(11) = 7.88, *p* = 0.0004, Cohen’s dz = 1.78) and in HICT (329.1 ± 83.4 mL of O_2_) compared to MICT (mean difference = 160.6 mL O_2_, 95% CI of difference [76.9–244.3]; t(11) = 9.04, *p* = 0.0002, Cohen’s dz = 1.89). No significant difference was found between HIIT and HICT (mean difference = –10.1 mL O_2_, 95% CI of difference [–73.6–93.9]; t(11) = –7.52, *p* = 0.95, Cohen’s d = 0.17).

### 3.3. Substrate Metabolism During and After MICT, HIIT and HICT

Table 2 presents a comparison of the oxidation rates of carbohydrates and lipids, EE, and the percentage of energy from carbohydrates and lipids during MICT, HIIT, and HICT during exercise and at 30 and 60 min after exercise.

During exercise, carbohydrate oxidation rate differed significantly across conditions (ANOVA: F(2,22) = 14.54, *p* < 0.0001, partial η^2^ = 0.57). Post hoc analyses revealed higher values in HIIT compared to MICT (mean difference = 11.4 mg·kg^−1^·min^−1^, 95% CI [3.9–18.9]; t(11) = 5.87, *p* = 0.002, Cohen’s dz = 1.69) and in HICT compared to MICT (mean difference = 15.6 mg·kg^−1^·min^−1^, 95% CI [8.1–23.1]; t(11) = 6.00, *p* < 0.0001, Cohen’s dz = 2.00). HICT did not differ significantly from HIIT carbohydrate oxidation rate (mean difference = 4.2 mg·kg^−1^·min^−1^, 95% CI [−3.3–11.69]; t(11) = 8.39, *p* = 0.35, Cohen’s dz = 0.82). Lipid oxidation rate did not differ significantly (ANOVA: F(2,22) = 2.49, *p* = 0.345, partial η^2^ = 0.19). Energy expenditure was comparable between conditions (ANOVA: F(2,22) = 0.84, *p* = 0.445, partial η^2^ = 0.07). The proportion of energy derived from carbohydrates was significantly greater in HIIT (74.6%) and HICT (78.5%) than in MICT (55.1%) (*p* < 0.01), with the inverse pattern observed for lipids (MICT: 44.9% vs. HIIT: 25.4% and HICT: 21.5%).

Thirty minutes after exercise, carbohydrate oxidation rate showed a main effect of condition (ANOVA: F(2,22) = 22.50, *p* < 0.0001, partial η^2^ = 0.70). HIIT (3.70 ± 1.04 mg·kg^−1^·min^−1^) and HICT (4.06 ± 1.03 mg·kg^−1^·min^−1^) were both significantly higher than MICT (1.42 ± 0.58 mg·kg^−1^·min^−1^) (HIIT vs. MICT: mean difference = 2.27, 95% CI [1.2–3.3]; t(11) = 17.4, *p* = 0.001, Cohen’s dz = 2.14; HICT vs. MICT: mean difference = 2.64, 95% CI [1.6, 3.7]; t(11) = 5.27, *p* < 0.0001, Cohen’s dz = 2.51). Lipid oxidation rate also differed (ANOVA: F(2,22) = 8.24, *p* < 0.0001, partial η^2^ = 0.43), with HIIT (1.08 ± 0.41 mg·kg^−1^·min^−1^) and HICT (1.20 ± 0.24 mg·kg^−1^·min^−1^) significantly greater than MICT (0.61 ± 0.20 mg·kg^−1^·min^−1^) (HIIT vs. MICT: mean difference = 0.47, 95% CI [0.20–0.74]; t(11) = 3.81, *p* = 0.007, Cohen’s dz = 1.1; HICT vs. MICT: mean difference = 0.60, 95% CI [0.35–0.83]; t(11) = 5.64, *p* < 0.0001, Cohen’s dz = 1.6). At 30 min post-exercise, EE differed significantly between conditions (ANOVA: F(2,22) = 21.21, *p* < 0.0001, partial η^2^ = 0.67). Post hoc analyses revealed higher EE in HIIT compared to MICT (mean difference = 13.4 cal·kg^−1^·min^−1^, 95% CI [7.9–18.9]; t(11) = 5.41, *p* = 0.0002, Cohen’s dz = 2.03) and in HICT compared to MICT (mean difference = 15.8 cal·kg^−1^·min^−1^, 95% CI [10.3, 21.4]; t(11) = 6.54, *p* < 0.0001, Cohen’s dz = 2.70). No significant difference was observed between HIIT and HICT (mean difference = −2.5, 95% CI [–6.5, 1.6]; t(11) = −1.29, *p* = 0.43, Cohen’s dz = −0.37). Substrate contribution analysis indicated a greater proportion of carbohydrates in HIIT (60.8%) and HICT (59.8%) compared to MICT (50%), with the opposite pattern observed for fat.

At sixty minutes after exercise, carbohydrate oxidation rate remained significantly different between conditions (ANOVA: F(2,22) = 17.90, *p* = 0.0002, partial η^2^ = 0.63). Post hoc analyses revealed greater values in HIIT compared to MICT (mean difference = 1.40 mg·kg^−1^·min^−1^, 95% CI [0.6–2.1]; t(11) = 4.78, *p* = 0.0005, Cohen’s dz = 2.04) and in HICT compared to MICT (mean difference = 1.68 mg·kg^−1^·min^−1^, 95% CI [0.88–2.4]; t(11) = 6.24, *p* < 0.0001, Cohen’s dz = 2.14). No significant difference was observed between HIIT and HICT (mean difference = 0.27 mg·kg^−1^·min^−1^, 95% CI [–0.5–1.0]; t(11) = 1.29, *p* = 0.222, Cohen’s dz = 0.34).

Lipid oxidation also differed significantly (ANOVA: F(2,22) = 17.70, *p* < 0.0001, partial η^2^ = 0.62). Both HIIT (mean difference vs. MICT = 0.74 mg·kg^−1^·min^−1^, 95% CI [0.3–1.1]; t(11) = 6.14, *p* < 0.0001, Cohen’s dz = 1.67) and HICT (mean difference vs. MICT = 0.90 mg·kg^−1^·min^−1^, 95% CI [0.5, 1.3]; t(11) = 7.23, *p* < 0.0001, Cohen’s dz = 2.67) were significantly higher than MICT. No significant difference was found between HIIT and HICT (mean difference = 0.14 mg·kg^−1^·min^−1^, 95% CI [–0.2, 0.5]; t(11) = 0.94, *p* = 0.367, Cohen’s dz = 0.27).

EE also differed significantly between conditions (ANOVA: F(2,22) = 12.59, *p* < 0.001, partial η^2^ = 0.65). HIIT elicited higher EE compared to MICT (mean difference = 12.3 cal·kg^−1^·min^−1^, 95% CI [6.9, 17.7]; t(11) = 4.94, *p* = 0.0003, Cohen’s dz = 1.43) and HICT was also greater than MICT (mean difference = 14.7 cal·kg^−1^·min^−1^, 95% CI [9.2, 20.2]; t(11) = 6.37, *p* < 0.0001, Cohen’s dz = 1.84). No difference was detected between HIIT and HICT (mean difference = 2.4 cal·kg^−1^·min^−1^, 95% CI [–2.3, 7.1]; t(11) = 1.08, *p* = 0.304, Cohen’s dz = 0.31).

The percentage of energy derived from carbohydrates was similar for MICT (46.22%), HIIT (45.55%), and HICT (45.76%). Finally, the percentage of energy from lipids did not differ significantly between MICT (53.78%), HIIT (54.45%), and HICT (54.24%). These results suggest that high-intensity training protocols (HIIT and HICT) promote greater carbohydrate utilization during exercise than does MICT. In addition, HIIT and HICT training sessions presented greater EE, as well as higher carbohydrate and lipid oxidation 60 min after the training sessions.

### 3.4. Total Substrate Metabolism During MICT, HIIT and HICT

Table 3 presents the cumulative substrate metabolism during the 60 min post-exercise recovery. Total lipid oxidation rate did not differ significantly between conditions (ANOVA: F(2,22) = 1.14, *p* = 0.338, partial η^2^ = 0.09). Post hoc comparisons confirmed no significant differences in lipid oxidation rate between MICT (5.16 ± 1.68 mg·kg^−1^·min^−1^), HIIT (5.77 ± 2.26 mg·kg^−1^·min^−1^), and HICT (5.81 ± 1.47 mg·kg^−1^·min^−1^), nor in total lipid-derived energy output (MICT: 46.40 ± 15.10 cal·kg^−1^·min^−1^; HIIT: 51.90 ± 20.36 cal·kg^−1^·min^−1^; HICT: 52.29 ± 13.26 cal·kg^−1^·min^−1^; all *p* > 0.05).

In contrast, total carbohydrate oxidation rate differed significantly between conditions (ANOVA: F(2,22) = 17.67, *p* = 0.0006, partial η^2^ = 0.60). HIIT elicited higher cumulative carbohydrate oxidation compared to MICT (mean difference = 15.1 mg·kg^−1^·min^−1^, 95% CI [5.0, 25.2]; t(11) = 3.28, *p* = 0.008, Cohen’s dz = 1.38), and HICT was also greater than MICT (mean difference = 19.9 mg·kg^−1^·min^−1^, 95% CI [10.1, 29.7]; t(11) = 4.50, *p* = 0.0004, Cohen’s dz = 2.21). No significant difference was found between HIIT and HICT (mean difference = 4.9 mg·kg^−1^·min^−1^, 95% CI [−4.0, 13.8]; t(11) = 1.18, *p* = 0.26, Cohen’s dz = 0.34). A similar pattern was observed for total carbohydrate energy output. HIIT exceeded MICT (mean difference = 60.3 cal·kg^−1^·min^−1^, 95% CI [20.1, 100.5]; t(11) = 3.30, *p* = 0.007, Cohen’s dz = 0.95), as did HICT compared to MICT (mean difference = 79.8 cal·kg^−1^·min^−1^, 95% CI [41.0, 118.6]; t(11) = 4.61, *p* = 0.0004, Cohen’s dz = 1.33). No significant difference was observed between HIIT and HICT (mean difference = 19.5 cal·kg^−1^·min^−1^, 95% CI [–15.8, 54.8]; t(11) = 1.16, *p* = 0.268, Cohen’s dz = 0.34).

### 3.5. Association Between Total Carbohydrate and Lipid Oxidation During Exercise and Post-Exercise Lipid Utilization

**Figure 3:**
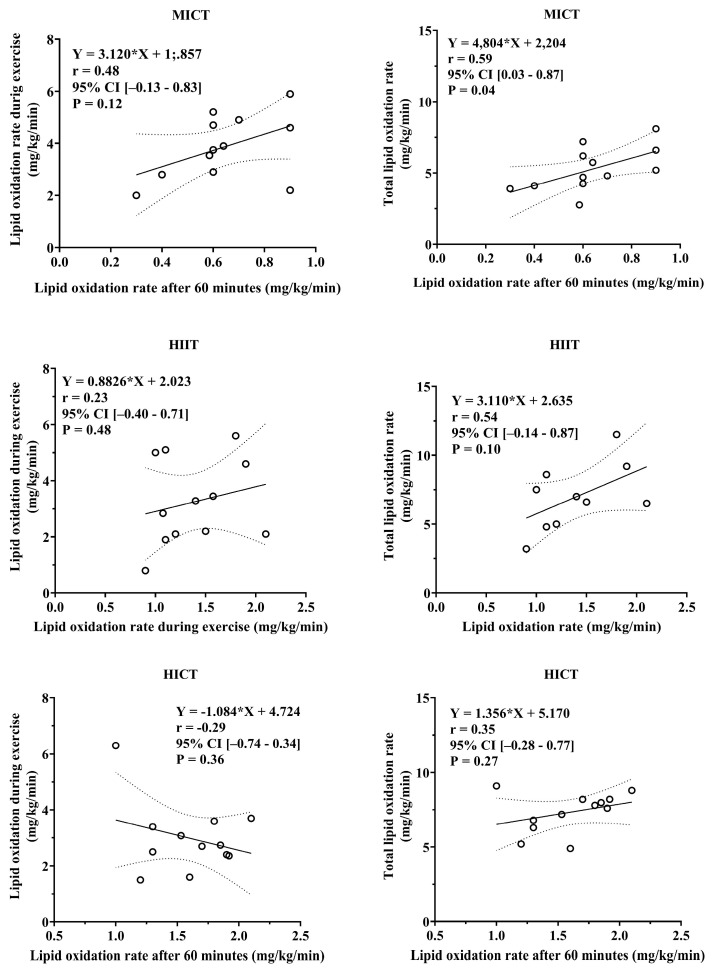
Pearson’s correlation coefficient between total lipid oxidation rate (**left**) and total energy production from lipid oxidation (**right**) at the end of the MICT, HIIT, and HICT training sessions, with a total lipid oxidation rate of 60 after the training sessions.

Figure 3 illustrates the Pearson correlation coefficient applied to quantify the association between the total lipid oxidation rate and total energy production from lipid oxidation during the MICT, HIIT, and HICT sessions, and the total lipid oxidation rate after 60 min. Thus, the total lipid oxidation rate and total energy production from lipid oxidation during the MICT, HIIT, and HICT sessions presented a weak-to-moderate correlation with the total lipid oxidation rate 60 min after the training sessions.

**Figure 4:**
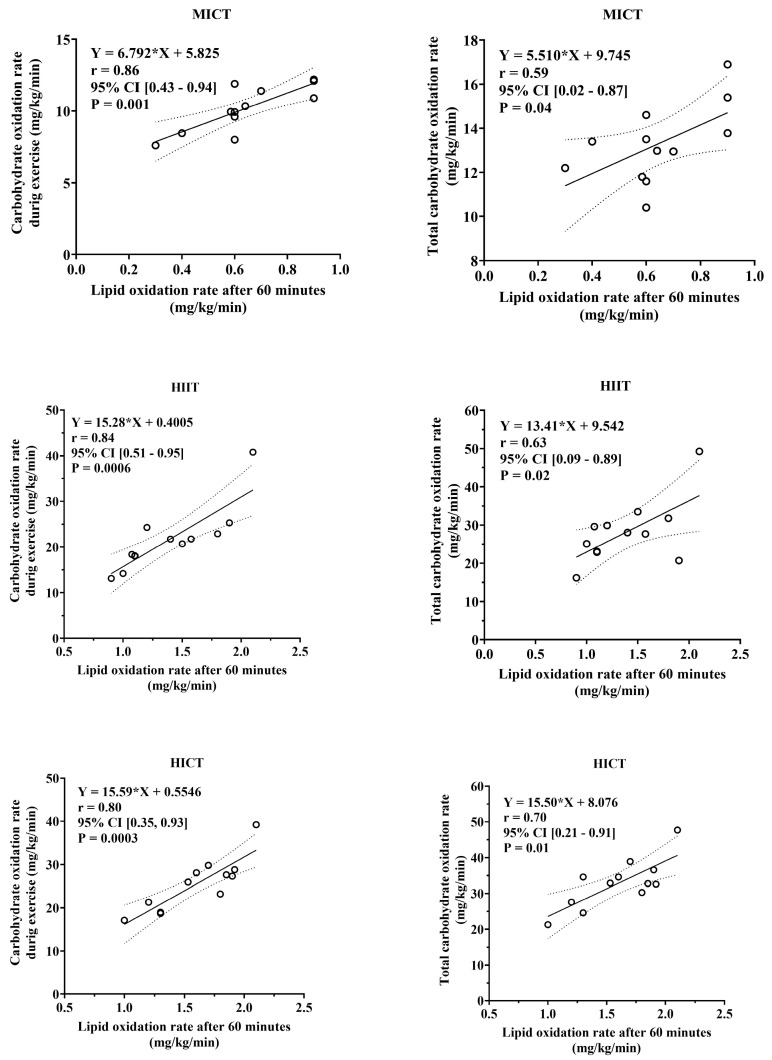
Pearson correlation coefficient between carbohydrate oxidation rate during exercise (**left**) and total carbohydrate oxidation rate (**right**) at the end of the MICT, HIIT, and HICT with the total lipid oxidation rate 60 min after the training sessions.

Additionally, Figure 4 shows the Pearson correlation coefficient applied to quantify the association between the total carbohydrate oxidation rate and total energy production from carbohydrate oxidation during the MICT, HIIT, and HICT sessions in relation to the total lipid oxidation rate after 60 min. Based on these results, we observed that the total lipid oxidation rate 60 min after the training sessions strongly correlated with the total carbohydrate oxidation rate and total energy production from carbohydrate oxidation rate during the MICT (r = 0.86, *p* = 0.001), HIIT (r = 0.84, *p* = 0.0006), and HICT (r = 0.80, *p* = 0.003) sessions. Thus, we can support the rationale that the total lipid oxidation rate after exercise is dependent on both the total carbohydrate oxidation rate and the total energy production from carbohydrate oxidation during training sessions.

## 4. Discussion

The aim of this study was to compare post-exercise energy expenditure (EE) and substrate mobilization following isocaloric bouts of moderate-intensity continuous training (MICT), high-intensity interval training (HIIT), and high-intensity Circuit training (HICT). Our main findings revealed significant differences in excess post-exercise oxygen consumption (EPOC) and substrate oxidation rates between the training protocols at 30 and 60 min post-exercise. Specifically, both HIIT and HICT demonstrated higher EPOC values, higher carbohydrate oxidation rates, and enhanced lipid oxidation than MICT despite similar total EE during the exercise sessions. These findings provide important insights into the metabolic effects of exercise intensity and modality on postexercise recovery.

Our findings are consistent with the well-documented intensity-dependent model of substrate utilization. During low-to-moderate intensities, fat plays a greater role as an energy source, while at higher intensities carbohydrates predominate due to increased glycolytic flux. Importantly, despite the suppression of fat oxidation during the exercise itself, recovery metabolism relies heavily on fat utilization, highlighting the significant contribution of lipids to post-exercise energy expenditure [25].

Excess post-exercise oxygen consumption (EPOC) is significantly influenced by factors such as exercise intensity, duration, and the volume of muscle recruitment [7]. Both the HIIT and HICT protocols are characterized by short, intense activity bouts with minimal rest periods, leading to substantial oxygen deficits during exercise and heightened reliance on anaerobic energy systems. Therefore, it is necessary to increase post-exercise oxygen uptake to restore homeostasis, including glycogen replenishment, lactate removal, hormonal rebalancing, and muscle tissue repair, thereby resulting in prolonged EPOC [25]. Conversely, MICT, which involves steady-state activity, induces a smaller oxygen deficit during exercise, leading to a lower EPOC magnitude.

Studies have demonstrated that higher exercise intensities elicit a more substantial EPOC response than lower intensities. For instance, research indicates that exercise intensities at or above 50–60% of VO_2_max are required to induce prolonged EPOC, with a curvilinear relationship observed between exercise intensity and EPOC magnitude. In this study, EPOC values were significantly higher in HIIT and HICT than in MICT over the 60 min post-exercise period [26]. This greater EPOC magnitude in HIIT and HICT aligns with findings from previous studies [9,10] that suggest that higher exercise intensities and reduced rest intervals significantly enhance post-exercise metabolic activity.

The intensity and nature of the preceding exercise significantly influenced energy expenditure (EE) during the post-exercise period. In our study, 30 min post-exercise, EE was notably higher in high-intensity circuit training (HICT) and high-intensity interval training (HIIT) than in moderate-intensity continuous training (MICT). This elevated EE persisted for 60 min, with HICT and HIIT maintaining superior caloric expenditure compared to MICT. These findings align with those of previous research, indicating that higher exercise intensities stimulate greater energy demands during recovery, resulting in elevated EE [26].

The elevated post-exercise EE observed in the HIIT and HICT groups could be attributed to several physiological mechanisms. High-intensity exercise depletes muscle glycogen stores more extensively, necessitating increased oxygen uptake during recovery for glycogen resynthesis [27]. Lactate accumulation during intense exercise requires post-exercise oxidation, which contributes to a sustained increase in EE. The greater disruption of homeostasis induced by high-intensity workouts also leads to increased hormonal responses, such as elevated catecholamine levels, which further enhances the metabolic rate during recovery [28].

Additionally, a recent study published by Ulupınar et al. [29] demonstrated that with an increase in the number of sprints during HIIT sessions, there was a reduction in the contribution of the phosphagen system and an increase in its dependence on glycolytic and oxidative systems. This generates a greater demand for oxygen in the post-exercise period, as the aerobic and glycolytic systems continue to replenish energy stores (phosphocreatine and glycogen) and remove metabolites, such as lactate. This phenomenon is closely linked to excess post-exercise oxygen consumption (EPOC), which is one of the main mechanisms of increased EE after intense activity [29].

In contrast, MICT, which is characterized by steady-state activity, induces a smaller oxygen deficit and less metabolic disturbance, resulting in lower EPOC and reduced post-exercise EE. Although MICT is effective in improving aerobic capacity and endurance, it may not elicit the same magnitude of post-exercise metabolic benefits as higher-intensity protocols. These findings underscore the efficacy of high-intensity training modalities such as HIIT and HICT in enhancing post-exercise EE [29,30]. By incorporating such protocols, individuals can achieve greater energy expenditure during recovery, which may be beneficial for weight management and metabolic health. However, it is essential to consider individual fitness levels and recovery capacities when designing exercise programs, because high-intensity workouts may not be suitable for everyone.

During the exercise sessions, the carbohydrate oxidation rates were significantly higher in HICT and HIIT than in MICT. These results are expected, as high-intensity protocols rely heavily on anaerobic glycolysis and the rapid breakdown of muscle glycogen to meet high-energy demands [7]. In contrast, lipid oxidation rates during exercise were similar across all protocols, reflecting the suppression of lipolysis and fat oxidation at higher exercise intensities due to increased lactate production and type II muscle fiber recruitment [30].

In the post-exercise phase, the substrate oxidation pattern significantly shifted. Thirty minutes after exercise, carbohydrate oxidation rates remained elevated in both HIIT and HICT compared to MICT. This trend persisted 60 min post-exercise, with carbohydrate oxidation remaining significantly higher in HIIT and HICT than in MICT. These findings replicate previous findings, which showed that greater muscle glycogen depletion during high-intensity HIIT and HICT exercise requires greater activation of metabolic pathways, such as gluconeogenesis and glycogen resynthesis in the liver and muscles, to replenish post-exercise glycogen stores [31]. This process involves an increase in oxygen consumption and metabolic rate, increasing EPOC, and prolonging EE after exercise [31].

The lipid oxidation rates also exhibited significant differences during the post-exercise period. At 30 min post-exercise, lipid oxidation rates were higher in the HIIT and HICT groups than in the MICT group. This pattern continued 60 min post-exercise, with HIIT and HICT maintaining superior lipid oxidation rates relative to MICT. These results are consistent with those reported by Jiang et al. [25], who found that a greater EPOC observed in high-intensity protocols was associated with enhanced fat oxidation during recovery.

During HIIT and HICT, anaerobic glycolysis predominates, and lipid oxidation is lower. However, in the post-exercise phase, the body must compensate for the depletion of energy substrates and progressively increase the mobilization of fatty acids for oxidation, which results in a higher rate of fat burning throughout recovery [25]. Therefore, the greater mobilization of lipids after HIIT and HICT compared to MICT suggests that exercise intensity has a direct impact on the prolongation of EPOC and the increase in EE during the recovery phase.

The relationship between carbohydrate and lipid oxidation during and after exercise was further supported by our correlation analysis, which revealed a strong association between total carbohydrate oxidation during exercise and lipid oxidation 60 min post-exercise. This suggests that the extent of carbohydrate depletion during high-intensity exercise directly influences the reliance of the body on lipid metabolism during recovery, highlighting the complementary role of these substrates in meeting energy demands [12,29,32,33,34].

Our findings are consistent with those of previous studies that demonstrated the metabolic benefits of high-intensity exercise. For example, Cunha et al. [35] reported that intermittent exercise protocols, such as HIIT, elicited greater EPOC values and post-exercise substrate oxidation than continuous protocols, such as MICT. Similarly, Greer et al. [36] showed that resistance training circuits and HIIT produced higher EPOC values than MICT, with no significant differences between the two high-intensity modalities. These studies have underscored the importance of exercise intensity in driving post-exercise metabolic responses.

More recently, Pilon et al. [10] compared isocaloric bouts of aerobic, resistance, and concurrent training, and found that aerobic exercise elicited higher EPOC values owing to its higher relative intensity. Our study builds on these findings by demonstrating that even under isocaloric conditions, the mode and intensity of exercise significantly influences post-exercise EE and substrate oxidation, with HIIT and HICT outperforming MICT in both metrics. While our findings provide important insights into the acute metabolic responses to different training modalities, it is important to acknowledge that this study was designed as an acute investigation. Therefore, any extrapolation to long-term weight management or chronic adaptations should be made with caution. Nevertheless, repeated bouts of higher-intensity exercise are mechanistically linked to greater cumulative EPOC, substrate oxidation, and energy expenditure, which may contribute to body weight and composition regulation over time. Future longitudinal research is required to confirm whether the acute advantages observed here translate into chronic metabolic benefits.

## 5. Conclusions

In conclusion, this study demonstrated that, while total EE during isocaloric bouts of MICT, HIIT, and HICT were similar, HIIT and HICT elicited significantly greater EPOC and substrate oxidation rates during the 30 and 60 min post-exercise periods. These findings highlight the acute metabolic advantages of high-intensity training protocols for optimizing post-exercise recovery and energy expenditure. Although our results suggest potential implications for long-term weight management, such benefits remain speculative and should be confirmed by longitudinal trials assessing chronic adaptations. Future studies are needed to expand these findings and to explore the long-term effects of these training modalities on metabolic health and body composition.

## Figures and Tables

**Table 1:** Participant Characteristics. Data are reported as the mean and standard deviation.

Variables	Healthy Trained Males (*n* = 12)
Age (years)	30.4 ± 4.0
Height (cm)	175.7 ± 8.5
Weight (kg)	82.3 ± 8.6
BMI (kg/m^2^)	26.8 ± 1.6
% FM	8.6 ± 1.9
VT (ml.kg.min^−1^)	30.6 ± 5.2
RCP (ml.kg.min^−1^)	43.2 ± 6.1
VO_2_max (ml.kg.min^−1^)	52.9 ± 6.3
VT (Watts)	175.0 ± 15.1
RCP (Watts)	239.6 ± 19.8
Max power (Watts)	289.6 ± 24.9
FC max (bpm)	190.7 ± 5.5

**Table 2:** Substrate metabolism during MICT, HIIT, and HICT at different periods.

Period		Carbohydrate Oxidation Rate (mg/kg/min)	Lipid Oxidation Rate (mg/kg/min)	EE (cal/kg/min)	Percentage of Energy from Carbohydrate (%)	Percentage of Energy from Lipid (%)
During exercise	MICT	10.34 ± 1.77	3.90 ± 1.43	76.4 ± 11.01	55.06 ± 12.57	44.94 ± 12.57
	HIIT	21.73 ± 8.50 **	3.28 ± 1.77	116.44 ± 35.97 **	74.62 ± 12.31 *	25.38 ± 12.31 *
	HICT	25.95 ± 6.75 ****	3.08 ± 1.46	131.47 ± 26.13 **	78.49 ± 10.88 **	21.51 ± 10.88 **
30 after exercise	MICT	1.42 ± 0.58	0.61 ± 0.20	11.19 ± 3.71	50.31 ± 7.32	49.69 ± 7.32
	HIIT	3.70 ± 1.04 ****	1.08 ± 0.41 **	24.57 ± 7.00 ***	60.79 ± 8.06 *	39.21 ± 8.06 *
	HICT	4.06 ± 1.03 ****	1.20 ± 0.24 ***	27.03 ± 6.16 ***	59.77 ± 3.76 **	40.23 ± 3.75 **
60 after exercise	MICT	1.25 ± 0.41	0.64 ± 0.22	10.63 ± 3.48	46.22 ± 4.24	53.78 ± 4.24
	HIIT	2.57 ± 0.81 ***	1.42 ± 0.43 ***	22.94 ± 6.61 ***	45.55 ± 3.85	54.45 ± 3.85
	HICT	2.89 ± 0.78 ****	1.54 ± 0.35 ****	25.36 ± 5.64 ****	45.76 ± 3.83	54.24 ± 3.83

* *p* = 0.01 vs. MICT; ** *p* = 0.002 vs. MICT; *** *p* = 0.0005 vs. MICT and **** *p* < 0.0001 vs. MICT.

**Table 3:** Total substrate metabolism during MICT, HIIT, and HICT.

Total Substrate Metabolism	MICT	HIIT	HICT
Total lipid oxidation rate (mg/kg/min)	5.16 ± 1.68	5.77 ± 2.26	5.81 ± 1.47
Total lipid energy output (cal/kg/min)	46.40 ± 15.10	51.90 ± 20.36	52.29 ± 13.26
Total carbohydrate oxidation rate (mg/kg/min)	12.98 ± 2.33	28.05 ± 9.69 **	32.92 ± 8.01 ***
Total carbohydrate energy output (cal/kg/min)	51.93 ± 9.33	112.19 ± 38.75 **	131.68 ± 32.06 ***

** *p* = 0.008 vs. MICT and *** *p* = 0.0004 vs. MICT.

## Data Availability

The raw data supporting the conclusions of this article will be made available by the authors upon request.

## Abbreviations

The following abbreviations are used in this manuscript:

AP	Average power
ATP	Adenosine triphosphate
BMI	Body mass index
BMR	Basal metabolic rate
CPET	Cardiopulmonary exercise test
EE	Energy expenditure
EPOC	Excess post-exercise oxygen consumption
FM	Fat mass
HICT	High-intensity circuit training
HIIT	High-intensity interval training
HR	Heart rate
kJ	kilojoules
LBM	Lean body mass
MICT	Moderate-intensity continuous training
One-Way	Unidirectional analysis of variance
ANOVA	
PAR-Q	Physical activity readiness questionnaire
PO	Power output
RCP	Respiratory compensation point
RER	Respiratory exchange ratio
RPE	Rating of perceived exertion
RT	Resistance training
SD	standard deviation
Two-Way	Bidirectional analysis of variance
ANOVA	
UNIVERSO	Salgado de Oliveira University
VCO2	Carbon dioxide production
VE/VCO_2_	Ventilatory equivalent for CO_2_
VE/VO_2_	Ventilatory equivalent for O_2_
VO_2_	Oxygen consumption
VT	Ventilatory threshold
